# Supplementary material for: Splice-Junction-Based Mapping of Alternative Isoforms in the Human Proteome
Source: Cell Rep. Author manuscript; Available in PMC 2020 Jan 15. (PMC6961840; doi:10.1016/j.celrep.2019.11.026)
Supplement: 2 [file NIHMS1546469-supplement-2.zip › DF1/PXD009737/TestisLysC/TestisLysC_8_MTMR12_ERAVMHQRQLSLPLTQSK.pdf]

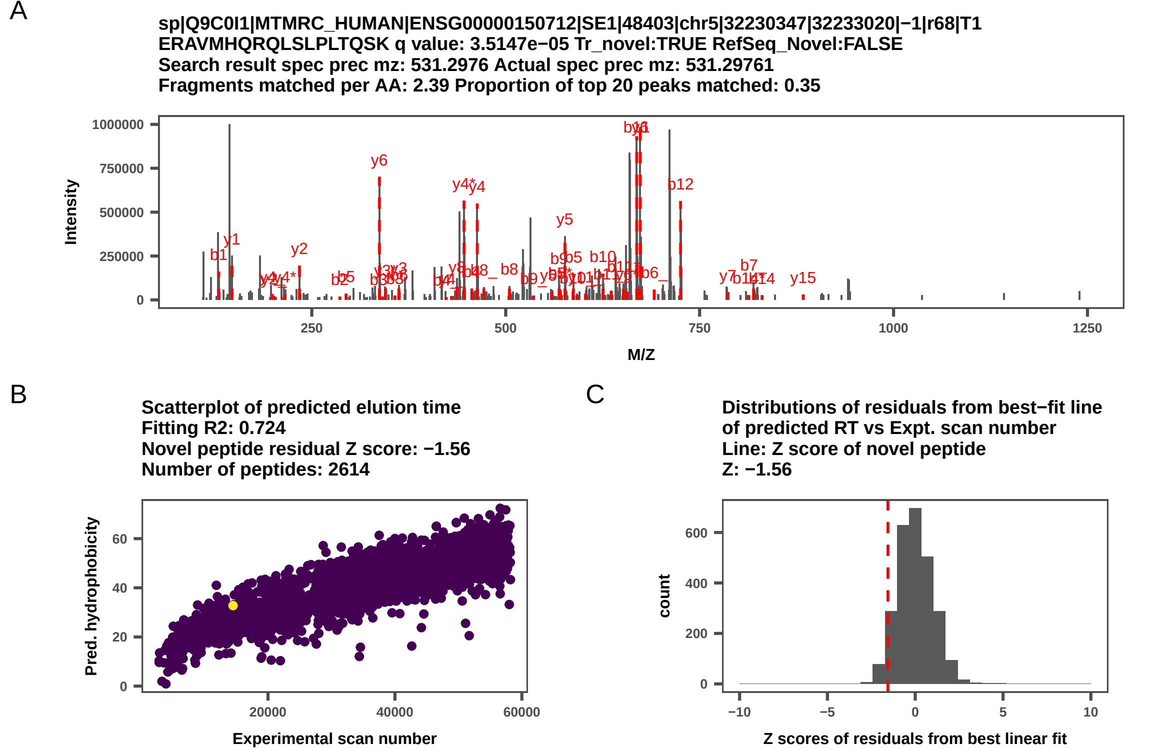
A
sp|Q9C0I1|MTMRC_HUMAN|ENSG00000150712|SE1|48403|chr5|32230347|32233020|−1|r68|T1
ERAVMHQRQLSLPLTQSK q value: 3.5147e−05 Tr_novel:TRUE RefSeq_Novel:FALSE
Search result spec prec mz: 531.2976 Actual spec prec mz: 531.29761
Fragments matched per AA: 2.39 Proportion of top 20 peaks matched: 0.35
Intensity
1000000
750000
500000
250000
0
y6
y4*
y4
b12
y5
y1
b1
y2
b10
b8
b7
y15
250
500
750
1000
1250
M/Z
B
Scatterplot of predicted elution time
Fitting R2: 0.724
Novel peptide residual Z score: −1.56
Number of peptides: 2614
Pred. hydrophobicity
0
20
40
60
20000
40000
60000
Experimental scan number
C
Distributions of residuals from best−fit line
of predicted RT vs Expt. scan number
Line: Z score of novel peptide
Z: −1.56
count
0
200
400
600
−10
−5
0
5
10
Z scores of residuals from best linear fit
